# DNA-PK hyperactivation occurs in deletion 11q chronic lymphocytic leukemia and is both a biomarker and therapeutic target for drug-resistant disease

**DOI:** 10.1038/s41408-022-00781-8

**Published:** 2023-01-27

**Authors:** Sara E. F. Kost, Ali Saleh, Shek H. Yuan, Bozena Kuzio, Spencer B. Gibson, Lin Yang, Versha Banerji, James B. Johnston, Sachin Katyal

**Affiliations:** 1 CancerCare Manitoba Research Institute, CancerCare Manitoba, Winnipeg, MB R3E 0V9 Canada; 2 Department of Pharmacology and Therapeutics, Max Rady College of Medicine, Rady Faculty of Health Sciences, University of Manitoba, Winnipeg, MB R3E 3P4 Canada; 3 Department of Oncology, University of Alberta, Edmonton, AB Canada; 4 Department of Internal Medicine, Max Rady College of Medicine, Rady Faculty of Health Sciences, University of Manitoba, Winnipeg, MB R3E 3P4 Canada; 5 Department of Biochemistry and Medical Genetics Max Rady College of Medicine, Rady Faculty of Health Sciences, University of Manitoba, Winnipeg, MB R3E 3P4 Canada

**Keywords:** Chronic lymphocytic leukaemia, Cell signalling, Preclinical research, Chronic lymphocytic leukaemia, B-cell receptor

Dear Editor,

While chemoimmunotherapy has been the standard treatment for chronic lymphocytic leukemia (CLL), it has been supplanted by targeted therapies, including the Bruton’s tyrosine kinase inhibitor, ibrutinib, and the bcl-2 inhibitor, venetoclax [1]. The prognosis and prediction of response to chemotherapy are intimately linked to the leukemic cell *IGHV* mutational status and fluorescent in situ hybridization abnormalities [2]. Patients with *IGHV* unmutated CLL have more aggressive disease with a preponderance of deletion 17p13 (del 17p; loss of *TP53* gene) or del 11q22-23 (del 11q; loss of ataxia telangiectasia mutated (*ATM*) gene) cases.

Del 11q occurs in 20% of CLL patients and is associated with a short time to first treatment (TTFT) and early relapse following chemotherapy [3]. However, these patients have an excellent response to ibrutinib [4]. Two-thirds of patients have an isolated del 11q and one-third an *ATM* mutation on the other allele, which can produce an additional loss of ATM function [5, 6]. However, an isolated del 11q has biological effects as telomere length is extremely short in del 11q cells, even without an additional *ATM* mutation [7]. Moreover, the fraction of the cell population with del 11q is important, with shortening of TTFT only occurring when >25–58% of cells have the deletion [8, 9]. These data suggest that loss of one ATM gene can have biological effects.

To determine if ATM protein levels are altered in CLL samples with del 11q, we examined the levels of total and phosphorylated (activated) ATM (pATM^S1981^) and DNA-PK (pDNA-PK^S2056^) by western blot in 30 patient samples with varying numbers of cells with del 11q (from 0 to 91%, Fig. 1A, B and Tables S1 and S2). Materials and Methods are in Supplementary. Twelve cases did not have del 11q or del 17 p (0%), 4 cases had a low del 11q fraction (<50%) while 14 cases had had a high del 11q fraction (>50%). While the levels of DNA-PK and ATM protein varied, ATM levels and the pATM:ATM ratio decreased as the fraction of del 11q cells increased (*p* = 0.009). These changes likely represent a dose-dependent loss of ATM activity as the fraction of cells with a del 11q increases, as mutations on the other allele do not necessarily correlate with further loss of ATM function [6]. In contrast, the pDNA-PK:DNA-PK ratio increased as the fraction of del 11q cells increased (*p* = 0.023). Similarly, the ratio of activated DNA-PK relative to activated ATM (pDNA-PK:DNA-PK/pATM:ATM) increased significantly (*p* = 0.0001) as the fraction of del 11q cells (Fig. 1A–C and Table S2) increased. Thus, DNA-PK is activated in del 11q cases with a high fraction of del 11 cells, to compensate for loss of ATM.

**Fig. 1:**
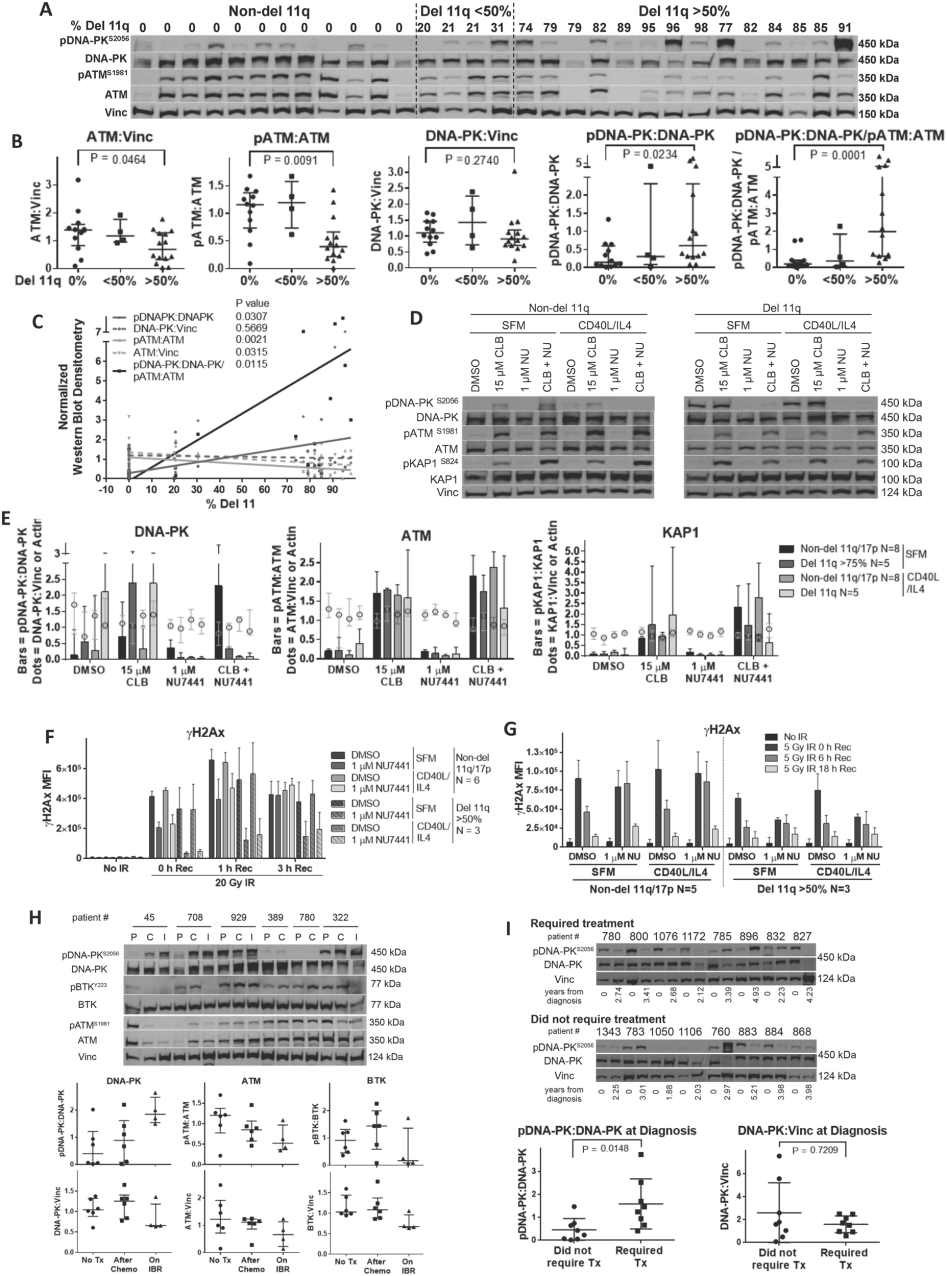
Impact of an increasing fraction of del 11q CLL cells on DNA-PK and ATM activation. **A** Western blot demonstrating the effect of a varying percentage of del 11 cells on protein levels of ATM, DNA-PK, and their activated phosphorylated derivatives, pDNA-PK and pATM. **B** Levels of DNA-PK, ATM, pATM:ATM, pDNA-PK:DNA-PK and pDNA-PK:DNA-PK/pATM:ATM in del 11q and non-del 11q cells (*p* values calculated using a Mann–Whitney test) and **C** relationship between the percentage of CLL cells with a deletion (del) 11q and the levels of DNA-PK, ATM and their phosphorylated derivatives (*p* values calculated using linear regression). Samples were all from untreated patients within 6 months of their FISH analysis. Vinc vinculin. Influence of chlorambucil and NU7441 treatment on DNA-PK activation and ATM function in del 11q CLL cells. **D** Representative western blots for (**E**) densitometry following ~18 h treatment of primary CLL cells in serum free media (SFM) or with CD40L/IL4 showing an increase in pDNA-PK in del 11q cells following 15 μM chlorambucil (CLB) treatment that was eliminated by combination with 1 μM NU7441 (NU), whereas pATM and pKAP1 levels were reduced in the del 11q cells with this co-treatment. Bars represent phospho-protein, dots represent total protein. Impact of DNA-PK inhibition γH2AX formation in del 11q CLL cells. CLL cells were treated for ~18 h with DMSO or 1 μM NU7441 in SFM alone or in the presence of CD40L/IL4 and irradiated during drug treatment with (**F**) 20 Gy or (**G**) 5 Gy and allowed to recover for 3, 1, or 0 h or 18, 6, or 0 h, respectively. Analyses of serine 139 phosphorylation of alternative histone H2AX (γH2AX) MFI (median fluorescence intensity) reveals that del 11q CLL cells accumulate less γH2AX compared to non-del 11q counterparts following IR-mediated induction of DNA strand breaks. While NU7441 co-treatment delays γH2AX formation in both CLL subtypes, del 11q cells are affected to a greater degree. DNA damage response (DDR) and B cell receptor (BCR) kinase activation in CLL patients following chemoimmunotherapy and short term ibrutinib treatment. **H** Western analysis of primary patient-matched CLL cells prior to treatment (P), following fludarabine, cyclophosphamide, rituximab (FCR) or chlorambucil/obinutuzumab chemotherapy (C) and four patient samples on ibrutinib (I; 45, 708, 929 and 322). Ratio of pDNA-PK:DNA-PK shows a progressive treatment-dependent increase of activated DNA-PK following chemotherapy and ibrutinib, coinciding with concomitantly-reduced ATM activation (pATM:ATM) and ibrutinib-mediated reduction in BTK activity (pBTK:BTK). Enhanced basal DNA-PK activation indicates eventual treatment. **I** Western analysis derived from these primary CLL patient cells shows that amongst patients who ultimately progressed to treatment (Tx), activated DNA-PK levels at diagnosis were significantly higher compared to those who did not require treatment after a similar time frame, while total DNA-PK levels were similar amongst the two cohorts. All plots in this figure are median ± interquartile range.

We then assessed the biological function of the DNA damage response (DDR) proteins in del 11q samples to determine if ATM function is lost and compensated for by an increase in DNA-PK activity. We thus compared non-del 11q and del 11q (>75% deletion) CLL samples following treatment with the DNA-cross-linking drug, chlorambucil, in the presence and absence of the DNA-PK inhibitor, NU7441. We also assessed the induction of phosphorylated KAP1 (pKAP1^S824^) as a measure of ATM function [6]. Chlorambucil increased DNA-PK activation to a 2.5-fold greater degree in del 11q samples, as compared to non-del 11q samples, and pDNA-PK formation was eliminated by NU7441 (Figs. 1D, E and S1 and Tables S1 and S3). pATM and pKAP1 levels were similarly increased by chlorambucil in all cases. pATM and pKAP1 increased further in non-del 11q samples following NU7441 but not in del 11q cells.

To evaluate the DDR, we compared the effects of NU7441 by γH2AX foci formation (measures DDR signaling at breaksites) and alkali comet assay (measures DNA strand breaks) in irradiated samples [10]. Del 11q cells (>50%) developed lower γH2AX levels compared to non-del 11q cells following irradiation (Fig. 1F and Table S1). Co-treatment with NU7441 delayed γH2AX formation and clearance, especially in del 11q samples (Fig. 1G and Table S1). Although ATM kinase is typically involved in γH2AX formation, DNA-PK is invoked with sub-optimal ATM function [11]. Furthermore, DNA strand break repair did not appear to be affected by del 11q status or DNA-PK activity (Fig. S2 and Table S1). Thus, ATM mediates DDR signaling in non-del 11q samples but these activities may be replaced by DNA-PK in del 11q cells [11].

DNA-PK activity may also be increased in CLL patients who have received prior chlorambucil [12]. We thus examined DNA-PK and ATM activation in six patients who received chemoimmunotherapy and then ibrutinib following relapse (Fig. 1H and Table S4). Activation of DNA-PK was significantly increased in four patients relapsing after chemoimmunotherapy (45, 708, 929, 322) paralleled by a decrease in activated ATM. In contrast, two patients had low pDNA-PK prior to therapy with little change following chemotherapy. DNA-PK activation increased further following ibrutinib, with a decline in activated ATM.

In contrast to ATM, enhanced DNA-PK supresses homologous recombination, induces non-homologous end-joining (NHEJ) and is associated with aggressive disease in multiple cancers [13]. To determine if this was true in CLL, pDNA-PK levels were compared in 16 new CLL patients, 8 requiring therapy in a median of 3 years, while 8 did not require treatment in this time period (Fig. 1I and Table S5). The baseline pDNA-PK:DNA-PK level was higher in those that required therapy suggesting that increased DNA-PK may explain the short TTFT in del 11q (*p* = 0.015).

To determine whether the elevated DNA-PK activity in del 11q cells influenced drug sensitivity, CLL samples were treated with NU7441. Interestingly, del 11q CLL cells were more sensitive to NU7441 than non-del 11q cases [14]. In total, 1 µM NU7441 decreased cell viability by 30% at 72 h (Fig. 2A and Table S1). In contrast, NU7441 had little activity against normal B (CD19+) and T (CD3+) cells, demonstrating that this is a tumor specific activity.

**Fig. 2:**
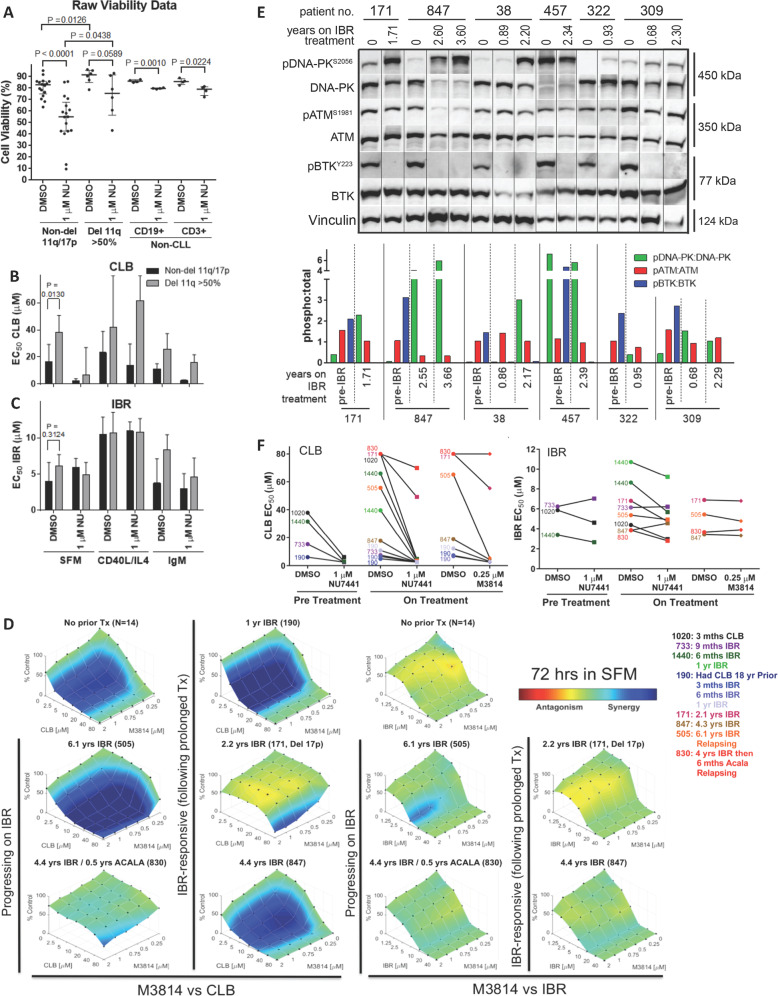
NU7441 is cytotoxic to CLL cells, but not normal B/T cells, and sensitizes CLL patient cells to chemotherapy. Primary CLL cells or PBMCs from non-CLL donors were treated with chlorambucil (CLB) or ibrutinib (IBR) for ~72 h alone or in the presence of 1 μM NU7441 (NU) in SFM, or co-treated with CD40L/IL4 or IgM and viability was measured by flow cytometry. **A** While CLL cell viability is reduced by NU7441, normal B (CD19+) and T (CD3+) cells are not. **B** Both del 11q and non-del 11q CLL cells treated with chlorambucil result in multi-fold lower EC_50_ values when co-treated with NU7441, while (**C**) EC_50_ values of ibrutinib are not affected by NU7441 co-treatment. Plots represent median ± interquartile range and *p* values were calculated using a paired *t*-test or a Mann–Whitney test. DNA-PK inhibition is synergistic with chlorambucil, even in CLL cells from patients on developing resistance to ibrutinib. **D** Combenefit synergy plots from CLL samples from patients who were treatment (Tx) naïve or on long-term ibrutinib treated ex vivo for ~72 h with combinations of chlorambucil or ibrutinib with M3814 in SFM, demonstrating marked synergy between M3814 and chlorambucil but not ibrutinib, even in patients who were receiving ibrutinib or were resistant to ibrutinib. Plots represent the degree of synergy (blue), additivity (green), or antagonism (red). Changes in DNA-PK and ATM activation are inversely correlated while CLL patients are on long-term ibrutinib. **E** Western blot of CLL cells taken prior to and while on long-term ibrutinib. pDNA-PK:DNA-PK levels increase in most patients while on long-term ibrutinib, pATM:ATM levels decrease in some patients and pBTK:BTK is eliminated by ibrutinib. DNA-PK inhibition is synergistic with chlorambucil, but not ibrutinib, in CLL patient cells following long-term ibrutinib treatment or resistance. **F** EC_50_ of chlorambucil and ibrutinib determined by flow cytometry (from 2D) following ~72 h treatment alone or with 1 μM NU7441 or 250 nM M3814 before or during clinical treatment of the patient, showing sensitization of cells to chlorambucil but not ibrutinib even when patients were receiving chlorambucil (1020), ibrutinib (733, 1440, 190, 171) or were refractory to ibrutinib (505, 830). Acala acalabrutinib.

Inhibition of DNA-PK has been shown to enhance drug sensitivity in CLL, so we assessed whether this was greater in del 11q cells (Fig. 2B, C and Tables S1 and S6) [14]. Six samples had >50% del 11q, 4 < 50% del 11q, 17 were non-del 11q and 1 del 17p. Following 72 h treatment, cell death was measured by Annexin V/7AAD staining and the drug concentration required to reduce cell viability by 50% (EC_50_) was measured. Samples with >50% del 11q cells were more resistant to chemotherapy than non-del 11q patients (median chlorambucil EC_50_, 38.1 μM versus 15.8 μM for del 11q and non-del 11q, respectively; Fig. 2B, *p* = 0.013). In contrast, the median ibrutinib EC_50_ for del 11q and non-del 11q cells were similar, at 6.1 and 3.9 μM, respectively (Fig. 2C, *p* = 0.3124). However, NU7441 sensitized both del 11q and non-del 11q cells to the chemotherapies chlorambucil, fludarabine or bendamustine to a similar extent (Tables S1 and S6). Resistant cells were also sensitized, including a sample with del 17p. NU7441 did not sensitize CLL cells to ibrutinib or idelalisib. NU7441 produced little sensitization of normal B and T cells to chlorambucil (Tables S1 and S6).

As NU7441 is not for clinical use, we evaluated M3814 and CC-115 in CLL cells, as both agents are being evaluated clinically [13, 15]. Initially, CLL cells from two patients were treated with CC-115 (inhibitor of DNA-PK/mTOR/PI3K) or M3814 (inhibitor of DNA-PK; Fig. S3) and chlorambucil or ibrutinib. Synergy was greater between chlorambucil and M3814 than between chlorambucil and CC-115; however, ibrutinib showed synergy with CC-115 but not M3814 (Fig. S4), likely a result of the effect of CC-115 on mTOR/PI3K activity. Further studies with M3814 showed it to be strongly synergistic with chlorambucil in CLL cells from 14 untreated patients without del 17p (Figs. 2D and S5), independent of del 11q status or anti-IgM treatment.

In patients on long-term ibrutinib, we observed enhanced pDNA-PK levels over time, which inversely correlated with pATM levels (Fig. 2E and Table S7). Importantly, sensitization between chlorambucil and M3814 was observed in cells from three of five patients on long-term ibrutinib (505, 190, and 847) including one (505) who was resistant to ibrutinib (Fig. 2D, F). Synergy increased throughout 1 year of ibrutinib treatment in patient 190 (Fig. S3). The combination of ibrutinib with M3814 in cells from patients on long-term ibrutinib showed additive or slightly synergistic cytotoxicity in three of four patients (505, 830, and 847) while the del 17p sample (171) showed antagonism (Fig. 2D, F).

These studies show that DNA-PK is activated and responsible for DDR in del 11q CLL cells, and this may explain the short TTFT and resistance to chemotherapy, but not ibrutinib, in these patients. DNA-PK is not activated by disease duration or progression but increases following ibrutinib and chemotherapy, suggesting that it is an effect of the DNA damage signaling response/NHEJ. Del 11q cells were more sensitive to DNA-PK inhibition than non-del 11q cells or normal lymphocytes, but inhibition sensitized all CLL types (but not normal lymphocytes) to chemotherapy, including those that were both chemotherapy- and ibrutinib-resistant. Hyperactivated DNA-PK is associated with aggressive disease in a variety of hematologic and solid tumors; likely reflecting enhanced mutation-prone NHEJ activity with genomic instability [13]. Thus, combining M3814 with chemotherapy may be a useful approach for treating multidrug-resistant CLL patients.

## Supplementary information

Supplementary Material

## Data Availability

Please e-mail the corresponding author for any data requests.
