# Supplementary material for: DNA-PK hyperactivation occurs in deletion 11q chronic lymphocytic leukemia and is both a biomarker and therapeutic target for drug-resistant disease
Source: Blood Cancer J. 2023 Jan 27;13(1):20. doi: 10.1038/s41408-022-00781-8 (PMC9883251; doi:10.1038/s41408-022-00781-8)
Supplement: Supplementary file 1 — Supplementary Material [file 41408_2022_781_MOESM1_ESM.pdf]

SUPPLEMENTAL MATERIALS AND METHODS

Patient samples and culture conditions

Informed consent was obtained from all participants, and the study was authorized by the human research ethics board at the University of Manitoba (REB HS2010:168 (HS10951), H2016:217 (HS19803), H2019:430 (HS23360), and H2021:306 (HS25134)). B cells were isolated from peripheral blood mononuclear cells (PBMC) from CLL patients or age-matched individuals without CLL (1). Freshly isolated cells were used for all experiments, unless otherwise specified and drug exposures were carried out in serum-free hybridoma media (SFM, Life Technologies, Carlsbad, CA). For the microenvironment simulation experiments, 50 ng/ml each of CD40L and IL4 (R&D Systems, Minneapolis, MN) or 10 µg/ml of AffiniPure F(ab")2 Fragment Goat Anti-Human IgM (IgM, Jackson Immunoresearch Labs) were added to cultures at the time of drug exposure. Drug exposures were carried out at 37°C and 5% CO_2 in a humidified atmosphere.

Drugs

Bendamustine, chlorambucil and fludarabine were purchased from MilliporeSigma (Oakville, ON), idelalisib, ibrutinib, IDE, CC-115 and NU7441 were purchased from Selleckchem (Houston, TX) and M3814 (preposertib) was purchased from ChemieTek (Indianapolis, IN). All drugs were reconstituted in DMSO (MilliporeSigma). DMSO alone was used as a negative control and, unless otherwise stated, for normalization.

FISH and *IGHV* mutational analysis

FISH analysis was carried out on fresh or stored cells, while *IGHV* mutational status was determined from RNA, as previously described (1).

Cytotoxicity by flow cytometry

For cytotoxicity studies, CLL cells were treated in 96-well plates with drug for 72 h in SFM with either DMSO or 1 μM NU7441. To simulate the microenvironment, cells were treated with CD40L/IL4 or anti-IgM. Treated cells were then mixed with either DMSO or 6 different concentrations of chlorambucil, fludarabine, bendamustine, idelalisib or ibrutinib to a maximum concentration of 80 μM, 20 μM, 160 μM, 80 μM or 40 μM, respectively. Concentration ranges for drug were optimized to ensure the exponential phase of the dose- response curve was obtained and the concentration of DMSO was constant between samples.

For synergy experiments, 6 concentrations of chlorambucil or ibrutinib were combined with 5 concentrations of M3814 or CC-115 (maximum concentration of 2 or 20 μM, respectively) and incubated for 72 h in SFM alone or with anti-IgM.

Cell death was determined using annexinV-FITC (AV) and 7-aminoactinomycin D (7AAD; both from BD Biosciences, San Jose, CA) (1). Cells were stained with AV/7AAD for 15 min and analyzed using a NovoCyte flow cytometer (ACEA Biosciences, San Diego, CA). Cells were considered alive when they were double-negative for AV and 7AAD. Non-CLL donor PBMCs were treated as above, with the exception that cells were only treated in SFM alone and with only 5 concentrations of either chlorambucil, fludarabine, bendamustine or ibrutinib due to cell number constraints. Post-drug incubation, cells were analyzed as above, except that anti-CD19-APC or anti-CD3-APC (BD Biosciences) were added in a triple stain with AV/7AAD to distinguish B and T cells, respectively. Isotype control antibodies were also run for CD19 (anti-mouse-IgG1κ, BD Biosciences) and CD3 (anti-mouse-IgG2aκ, BD Biosciences).

DNA damage analysis sample preparation

Two experiments were performed to study the DNA damage response to ionizing radiation (IR), one with shorter recovery times with higher IR and one with longer recovery times and lower IR. In both, cells were treated with either CD40L/IL4 or left unstimulated (SFM only) for the duration of the experiment, incubated in a 96-well U bottom plate, and non-irradiated cells were used as a negative control. In the first experiment, cells were immediately treated with DMSO or 1 μM NU7441 and allowed to incubate for ~18h. At 3h, 1h, or immediately before the end of the ~18h incubation, cells were irradiated with 20 Gy IR using a RS2000 Rad Source (Rad Source Technologies, Inc., Buford, GA), allowing for 3 h, 1 h, or no recovery. After the ~18 h, an aliquot of cells was removed to perform the comet assay and the remainder of the cells were used for γH2AX staining. In the second experiment, cells were treated with DMSO or 1 μM NU7441 and incubated for a total of ~18 h. Cells were exposed to 5 Gy IR at the beginning of the ~18 h treatment, or 6 h or immediately prior to the end of the drug treatment, allowing for ~18 h, 6 h, or no recovery. Cells were then used for γH2AX staining.

DNA damage analysis by γH2AX flow cytometry

γH2AX staining was performed as previously described (1) and analyzed by flow cytometry using a NovoCyte flow cytometer.

DNA damage analysis by Comet assay

The alkaline comet assay was performed as previously described (1,2) on 96-well slides (Trevigen, Gaithersburg, MD) and comets were visualized via SYBR green staining on a Cytation 5 (BioTek Instruments, Winooski, VT) (1).

Western blot analyses

For western blot analysis using untreated samples from specific time points, cell pellets or cryo-preserved cells were thawed and lysed immediately. Using fresh cells, primary CLL cells were treated with 250 nM or 500 nM M3814, 1 µM NU7441, 15 µM chlorambucil, or the combination of chlorambucil with M3814 or NU7441 for ~18 h prior to lysis.

Protein extracts were prepared using lysis buffer (50 mM Tris-HCl, 200 mM NaCl, 0.2% NP-40, 1% Tween-20 (v/v)) supplemented with 1 mM NaF, 1 mM sodium vanadate, 50 mM β-glycerophosphate, 2 mM PMSF and Complete protease inhibitor (Roche). Following quantification by Bradford assay (Bio-Rad), proteins (20 µg per lane) were separated through a Novex 4–12% (w/v) Bis-Tris SDS polyacrylamide gel (Life Technologies) and transferred onto nitrocellulose membrane (Bio-Rad). Blots were sequentially immunostained with the antibodies indicated in Supplementary Table S8. Blots were subsequently incubated with either horseradish peroxidase– conjugated goat anti-mouse (Bio-Rad, cat# 1706516, 1:2000) or goat anti-rabbit (Bio-Rad, cat# 1706515, 1:2000) and detected using Clarity chemiluminescence reagent (Bio-Rad) and imaged using a ImageQuant LAS 500 automated chemiluminescence imager. Rabbit anti-actin ab (Millipore-Sigma, cat# A2066, 1:2000) or mouse anti-vinculin ab (Millipore-Sigma, cat# A2066, 1:2000) were used as protein-loading controls. Cell treatments for Western analysis are in each respective figure legend. Blots were visualized using a LAS 500 Imager (GE Healthcare). Densitometry analysis of individual protein band signals was performed via ImageJ. Protein levels were first normalized to a loading control and any concomitant phospho-protein levels were normalized to their non-phosphorylated (total) counterparts.

Statistical and synergy analysis

Graphical representation and statistical analysis were performed using MS Excel and GraphPad Prism. To test the differences between non-del 11q and del 11q samples, a Mann Whitney test was performed. To determine the differences between stimulation methods or drug treatments, a paired T test was used with a 95% confidence interval. To determine the correlation between percentage of cells with del 11q and the levels of the DDR proteins, the Pearson correlation coefficient (r) was calculated and the p-value was determined using a two- tailed T test with a 95% CI. Drug synergy was assessed using Combenefit software (1,3). Combenefit plots represent the average difference in cell viability compared to that predicted to the single dose-response curves for each agent (blue-synergy, green-additive, red-antagonism). For all tests a p-value of <0.05 was considered significant.

References

1. Kost, Saleh, Mejia, Mostafizar, Bouchard, Banerji, et al. Transcriptional Modulation by Idelalisib Synergizes with Bendamustine in Chronic Lymphocytic Leukemia. Cancers (Basel) [Internet]. 2019 Oct 9;11(10):1519. Available from: https://www.mdpi.com/2072-6694/11/10/1519
2. Katyal S, Lee Y, Nitiss KC, Downing SM, Li Y, Shimada M, et al. Aberrant topoisomerase-1 DNA lesions are pathogenic in neurodegenerative genome instability syndromes. Nat Neurosci. 2014;17(6):813–21.
3. di Veroli GY, Fornari C, Wang D, Mollard S, Bramhall JL, Richards FM, et al. Combenefit: An interactive platform for the analysis and visualization of drug combinations. Bioinformatics. 2016;32(18):2866–8.

LEGENDS TO SUPPLEMENTARY TABLES

Table S1: Clinical data for samples used in Figures 1-2, Supplemental Tables S2, S3, S6 and Figures S1 and S2.

Table S2: Raw western blot densitometry from Figure 1A-C of untreated primary CLL cells within 6 months of when their FISH was determined. Relative values are shown as a heatmap with lower values represented in green and higher values represented in red.

Table S3: Raw western blot densitometry from Figure 1D-E and a del 17p primary CLL sample following ~18 h treatment with 15 μM chlorambucil (CLB) and/or 1 μM NU7441 in serum free media (SFM) alone or with CD40L/IL4. Relative values are shown as a heatmap with lower values represented in green and higher values represented in red.

Table S4: Clinical data for samples used in Figure 1H.

Table S5: Clinical data for samples used in Figure 1I.

Table S6: Raw EC_{50} values for Figure 2B-C following ~72 h drug treatment of CLL cells or non-CLL donor peripheral blood mononuclear cells (PBMCs; stained with CD19 or CD3 to distinguish B and T cells, respectively) with chemotherapy (chlorambucil (CLB), fludarabine (FLU), or bendamustine (BEN)) or targeted therapies (ibrutinib (IBR) or idelalisib (IDE)) in serum free media (SFM), CD40L/IL4 or IgM alone or in the presence of 1 μM NU7441. Relative values are shown as a heatmap with lower values represented in green and higher values represented in red.

Table S7: Clinical data for samples used in Figure 2D.

Table S8: Antibodies used for western blot analysis.

LEGENDS TO SUPPLEMENTARY FIGURES

Figure S1: Raw western blots from Figure 1D-E and a del 17p primary CLL sample following ~18 h treatment with 15 μM chlorambucil (CLB) and/or 1 μM NU7441 in serum free media (SFM) alone or with CD40L/IL4.

Figure S2: Impact of DNA-PK inhibition on DNA break formation in del 11q CLL cells. CLL cells were treated for ~18 h with DMSO or 1 μM NU7441 in SFM alone or in the presence of CD40L/IL4 and irradiated during drug treatment with 20 Gy and allowed to recover for 3, 1, or 0 h. Irrespective of DNA-PK inhibitor treatment or del 11q status, alkali comet assay analyses reveal no difference in DNA strand break accumulation or DNA repair activity. Plots are median +/- interquartile range.

Figure S3: (A) Western blot showing inhibition of DNA-PK by both NU7441 and M3814 after ~18 h incubation with one primary CLL sample (190). (B) Synergy plots of CLL cells from one

patient (190) of the combination of ~72 h *ex vivo* treatment with chlorambucil (CLB) and M3814 in SFM alone or in IgM prior to and 3, 6, and 12 months post clinical ibrutinib treatment of the patient showing increased synergy over clinical treatment. Plots represent the degree of synergy (blue), additivity (green), or antagonism (red). (C) Table of clinical data and EC_{50}s of chorambucil flowing ~72 h treatment alone or in the presence of 1 μM NU7441 or 0.25 or 0.5 μM M3814 using the same clinical time points as (B). Relative values are shown as a heatmap with lower values represented in green and higher values represented in red.

Figure S4: (A) Synergy plots of CLL cells from 2 patients (1020 and 1374) of the combination of 72 h *ex vivo* treatment with chlorambucil (CLB) or ibrutinib (IBR) and M3814 or CC-115 in SFM showing more synergy between chlorambucil and M3814 than CC-115 but the opposite with ibrutinib. Plots represent the degree of synergy (blue), additivity (green), or antagonism (red). (B) Table of clinical data and EC_{50}s of chlorambucil and ibrutinib flowing ~72 h treatment alone or in the presence of 1 μM NU7441 or 0.5 μM M3814 or CC-115. Relative values are shown as a heatmap with lower values represented in green and higher values represented in red.

Figure S5: (A) Table of clinical data and EC_{50}s of chlorambucil (CLB) and ibrutinib (IBR) flowing ~72 h treatment alone or in the presence of 1 μM NU7441 or 0.25 or 0.5 μM M3814 showing sensitization to CLB with DNA-PK inhibition even following clinical treatment. Relative values are shown as a heatmap with lower values represented in green and higher values represented in red. (B) Raw synergy plots of the no prior treatment CLL patient samples from Figure 2E primary following ~72 h treatment with chlorambucil or ibrutinib combined with M3814 in SFM alone or with IgM. Plots represent the degree of synergy (blue), additivity (green), or antagonism (red).

Table S1

Pt#	Sex	Age	*IGHV*	FISH (%)	Treatment (Yrs prior)	Figure(s)	Table
257	M	67	U	Tri 12 (61)	None	1A(#7),B-C,E; S1	S2(#7); S3
313	F	61	U	Normal	None	1A(#1),B-C	S2(#1)
368	M	64	M	Del 13q (93)	None	1F; S2	
389	M	59-60	U	Del 11q (85), Tri 12 (94)	None	1A(#29),B-C	S2(#29)
453	M	57	U	Tri 12 (68)	FCR (4.1)	2A-C	S6(#21)
472	F	60	U	Del 11q (95), 13q (97)	None	1A(#22),B-C	S2(#22)
535	M	79	M	Del 11q (21), 13q (93)	None	1A(#14),B-C	S2(#14)
598	F	57	U	Del 11q (85)	None	1A(#28),B-C	S2(#28)
651	F	90	M	Del 13q (91)	None	2A-C	S6(#19)
708	F	72	U	Del 11q (66), Del 13q (62)	FCR (3.8)	2A-C	S6(#24)
733	F	89	M	Del 13q (82)	None	2A-C	S6(#9)
735	M	47	M	Del 13q (10)	None	2A-C	S6(#11)
758	M	66	U	Del 11q (82), Del 13q (76)	None	1A(#26),B-C	S2(#26)
789	M	73	U	Del 11q (18), Tri 12 (44)	FLU (8.2), FCR (5.1)	2A-C	S6(#15)
833	F	58	M	Del 13q (86)	None	1A(#2),B-C	S2(#2)
834	M	63	M	Del 11q (79), Del 13q (66)	None	1A(#18),B-C	S2(#18)
867	M	95	U	Tri 12 (90)	None	1A(#8),B-C	S2(#8)
879	M	58	U	Del 11q (77), Del 13q (60)	None	1A(#25),B-C	S2(#25)
881	F	61	U	Del 11q (97), Del 13q (99)	FCR (3.4), FR (3.1)	2A-C	S6(#8)
936	M	77	M	Normal	None	1A(#9),B-C,G	S2(#9)
962	M	64-65	U	Del 13q (83)	None	1A(#3),B-C	S2(#3)
929	M	68	M	Del 11q (91), Del 13q (94)	None	1A(#30),B-C	S2(#30)
1010	M	60	U	Del 13q (92)	None	1A(#6),B-C	S2(#6)
1020	M	84	U	Del 11q (82), Del 13q (81)	None	1A(#20),B-C; 2A-C	S2(#20); S6(#23)
1033	F	74	U	Del 13q (86)	None	1A(#4),B-C	S2(#4)
1063	M	88	U	Del 11q (84), Tri 12 (81)	None	1A(#27),B-C	S2(#27)
1089	M	64	U	Normal	None	1E,F; 2A-C; S1; S2	S3; S6(#25)
1090	M	66	U	Del 11q (31)	None	1A(#16),B-C	S2(#16)
1093	F	78-79	M	Del 11q (96), Del 13q (74)	None	1E-G; 2A-C; S1; S2	S3; S6(#18)
1105	F	88	U	Del 11q (96)	None	1A(#23),B-C	
1114	F	94	M	Tri 12 (87)	None	2A-C	S6(#22)
1126	F	72	M	Del 13q (79)	None	1A(#12),B-C,D(#1),E,G; 2A-C; S1	S2(#12); S3; S6(#1)
1168	F	68	M	Tri 12 (92)	None	2C	
1172	M	61-63	U	Del 11q (98), Del 13q (98)	None	1A(#24),B-C,E; S1	S2(#24); S3
1173	F	71	U	Del 11q (79)	None	1A(#19),B-C,E-G; 2A-C; S1; S2	S2(#19); S3; S6(#27)
1177	F	83	N/A	Del 11q (20), Del 13q (40)	None	1A(#13),B-C	S2(#13)
1191	F	86-87	U	Tri 12 (68)	None	1E,F; 2A-C; S1; S2	S3; S6(#5)
1197	F	88	M	Del 13q (92)	None	1A(#5),B-C	S2(#5)
1221	M	79	M	Del 13q (17)	None	1G	
1235	M	67	N/A	Del 13q (11)	None	2A-C	S6(#13)
1258	F	85	U	Del 11q (10), Del 13q (68)	CLB (6.7), CLB/Pred (4.1), FR (1.7)	2A-C	S6(#10)
1262	M	86	M	Normal	None	1E,F; 2A-C; S1; S2	S3; S6(#4)
1297	M	50-51	U	Normal	None	1A(#10),B-C; 2A-C	S2(#10); S6(#17)
1301	F	52	M	Tri 12 (3), Del 13q (60)	None	2A-C	S6(#2)
1309	F	84	U	Del 17p (84)	None	S1	S6(#28)
1320	M	80	U	Tri 12 (60)	None	2A-C	S6(#3)
1356	M	63	M	Del 11q (74)	None	1A(#17),B-C	S2(#17)
1377	F	88-91	U	Del 11q (21), Del 13q (91)	Fig 1: None; Fig 3: CLB/Ob (2.96)	1A(#15),B-C; 2A-C	S2(#15); S6(#16)
1387	M	58	U	Del 13q (65)	None	1E,F S1; S2	S3
1406	F	84	U	Del 11q (98), Del 13q (49)	None	1A(#21),B-C,E; S1	S2(#21); S3
1410	F	81	U	Tri 12 (63)	None	1E; S1	S3
1413	F	71-74	U	Tri 11q (87), Del 13q (83)	None	1D(#2),E,G; 2A-C; S1	S3; S6(#26)
1419	F	80	U	Del 13q (71)	None	1A(#11),B-C,G; 2A-C	S2(#11); S6(#14)
1428	M	74	N/A	Del 11q (60), Del 13q (60)	None	1F; S2	
1440	M	69	U	Del 11q (27)	None	2A-C	S6(#30)
1441	M	84	M	Normal	None	1E,F; 2A-C; S1; S2	S3; S6(#6)
1453	M	78	U	Del 13q (50)	None	2A-C	S6(#12)
1505	F	58	U	Del 13q (94)	None	2A-C	S6(#7)
N130	F	67			None	2A	S6
N176	F	61			None	2A	S6
N181	F	61			None	2A	S6
N260	F	59			None	2A	S6

Pt, patient; N, Non-CLL Donor; M, mutated; U, unmutated; N/A, not available; Pred, prednisone; FR, FLU and rituximab; FCR, FLU, cyclophosphamide, R; Del, deletion; Tri, trisomy

Table S2

#	% Del 11q	pDNA-PK:DNA-PK	DNA-PK:Vinc	pATM:ATM	ATM/Vinc	pDNA-PK:DNA-PK/ pATM:ATM
1	0	0.64	0.51	0.43	0.09	1.48
2	0	0.05	0.96	0.69	1.40	0.07
3	0	0.18	1.46	1.26	1.41	0.15
4	0	0.65	1.68	1.68	1.63	0.39
5	0	0.08	1.46	1.03	1.37	0.08
6	0	0.46	1.02	1.15	1.10	0.40
7	0	0.34	1.18	1.44	1.45	0.24
8	0	0.07	0.96	1.17	1.29	0.06
9	0	0.01	1.73	1.40	3.18	0.01
10	0	1.34	0.75	0.88	0.73	1.52
11	0	0.10	1.23	1.30	2.07	0.08
12	0	0.02	0.45	0.09	0.30	0.27
13	20	0.03	1.02	1.09	1.32	0.03
14	21	0.35	2.39	0.62	0.93	0.57
15	21	0.26	1.84	1.67	1.92	0.16
16	31	2.98	0.63	1.30	1.03	2.28
17	74	2.03	0.95	0.94	0.91	2.16
18	79	0.29	1.42	0.46	1.35	0.62
19	79	0.31	0.23	0.42	0.03	0.73
20	82	1.02	1.45	0.57	1.26	1.80
21	89	0.94	0.71	0.23	0.04	4.10
22	95	0.39	1.11	0.01	0.43	28.70
23	96	8.28	0.61	1.43	0.47	5.80
24	98	0.81	1.09	0.27	1.41	3.01
25	77	3.19	0.86	0.37	0.37	8.53
26	82	0.09	0.71	0.14	0.18	0.61
27	84	0.39	0.98	0.55	0.95	0.70
28	85	0.09	3.03	0.18	0.39	0.48
29	85	0.32	0.72	1.20	1.77	0.27
30	91	6.73	0.84	0.34	1.23	20.06

Table S3

			pDNA-PK:DNA-PK													
	Patient		1126	257	1410	1441	1387	1089	1262	1191	1173	1093	1413	1406	1172	1309
Well	% Del 11q		0	0	0	0	0	0	0	0	79	85	87	89	98	Del 17
1	DMSO	SFM	0.07	0.12	0.14	0.02	1.16	1.81	0.25	0.10	0.50	0.55	1.38	1.93	0.08	0.81
2	15 µM CLB		1.35	0.51	0.57	0.45	1.95	2.81	0.53	0.87	2.66	1.84	2.39	4.89	1.16	1.44
3	1 µM NU7441		0.09	0.53	1.69	0.04	0.61	0.18	0.13	0.60	0.03	0.19	0.05	0.24	0.03	0.13
4	CLB + NU7441		4.15	6.32	19.66	0.89	1.06	0.38	1.23	3.36	0.34	0.43	0.09	0.35	0.11	0.51
5	DMSO	CD40L/IL4	0.33	10.79	0.15	0.02	1.89	2.04	0.21	0.23	1.92	2.67	2.12	0.63	3.79	2.39
6	15 µM CLB		1.14	0.31	0.31	0.35	0.24	2.30	0.21	0.75	1.79	4.09	2.38	1.68	2.48	2.93
7	1 µM NU7441		0.21	0.09	0.03	0.04	0.04	0.08	0.00	0.05	0.03	0.12	0.04	0.06	0.02	0.03
8	CLB + NU7441		0.01	0.11	0.02	0.06	0.08	0.12	0.01	0.14	0.03	0.20	0.10	0.05	0.20	0.08

			pATM:ATM													
	Patient		1126	257	1410	1441	1387	1089	1262	1191	1173	1093	1413	1406	1172	1309
Well	% Del 11q		0	0	0	0	0	0	0	0	79	85	87	89	98	Del 17
1	DMSO	SFM	0.22	0.25	0.16	0.07	0.21	0.26	0.09	0.31	0.47	0.07	0.22	0.66	0.15	0.24
2	15 µM CLB		1.62	1.38	2.44	1.75	1.53	1.65	1.79	2.52	1.88	1.80	1.81	0.79	1.60	1.85
3	1 µM NU7441		0.13	0.25	0.18	0.07	0.23	0.25	0.15	0.24	0.42	0.13	0.19	0.03	0.14	0.73
4	CLB + NU7441		2.65	1.35	2.81	1.87	2.72	2.17	2.04	2.15	1.53	2.02	1.74	0.07	2.35	1.82
5	DMSO	CD40L/IL4	0.09	2.37	0.08	0.11	0.13	0.07	0.06	0.25	0.74	0.22	0.40	0.81	0.13	0.41
6	15 µM CLB		1.79	1.39	1.98	1.52	1.72	1.52	1.59	2.98	1.43	1.78	1.58	3.90	1.21	1.90
7	1 µM NU7441		0.10	0.12	0.06	0.10	0.18	0.08	0.06	0.05	0.13	0.10	0.47	0.12	0.09	0.33
8	CLB + NU7441		2.82	1.77	2.68	2.27	2.50	2.19	2.24	3.18	1.22	1.32	1.92	0.37	3.44	2.32

			pKAP1:KAP1													
	Patient		1126	257	1410	1441	1387	1089	1262	1191	1173	1093	1413	1406	1172	1309
Well	% Del 11q		0	0	0	0	0	0	0	0	79	85	87	89	98	Del 17
1	DMSO	SFM	0.04	0.01	0.03	0.07	0.33	0.08	0.18	0.16	0.05	0.19	0.06	0.03	0.20	0.83
2	15 µM CLB		0.93	0.12	0.85	0.83	0.84	0.29	0.49	0.99	1.46	5.91	2.65	0.05	1.47	0.87
3	1 µM NU7441		0.11	0.01	0.36	0.20	0.37	0.06	0.06	0.22	0.11	0.04	0.05	0.01	0.10	0.49
4	CLB + NU7441		2.73	1.60	3.52	2.81	1.79	3.86	1.80	1.92	3.16	0.57	1.45	0.01	3.70	1.16
5	DMSO	CD40L/IL4	0.04	3.91	0.11	0.08	0.22	0.01	0.27	0.21	0.63	0.06	0.04	0.01	0.02	0.54
6	15 µM CLB		1.14	0.58	0.94	0.41	1.04	0.88	0.48	1.35	1.94	1.34	2.39	7.98	1.48	1.12
7	1 µM NU7441		0.04	0.01	0.09	0.05	0.22	0.01	0.03	0.13	0.32	0.05	0.02	0.01	0.04	0.46
8	CLB + NU7441		2.85	2.70	3.36	2.32	1.32	4.78	1.51	4.84	0.51	0.62	1.93	0.01	2.08	2.66

			DNA-PK:Vinc or Actin													
	Patient		1126	257	1410	1089	1262	1441	1387	1191	1173	1093	1413	1406	1172	1309
Well	% Del 11q		0	0	0	0	0	0	0	0	79	85	87	89	98	Del 17
1	DMSO	SFM	1.05	1.88	0.70	1.53	2.13	2.55	0.64	1.89	0.71	1.69	1.28	0.64	0.66	1.03
2	15 µM CLB		1.15	1.97	1.21	1.08	1.41	1.09	0.38	1.08	0.73	1.14	1.11	0.24	1.06	0.95
3	1 µM NU7441		0.95	1.59	0.70	0.99	1.00	1.25	1.21	1.10	0.96	1.00	0.92	0.10	1.61	1.07
4	CLB + NU7441		1.19	0.25	0.38	1.08	0.71	1.78	0.91	0.58	0.68	1.11	0.99	0.09	1.15	1.03
5	DMSO	CD40L/IL4	1.26	0.50	1.47	0.98	1.53	2.00	0.51	1.48	1.49	1.06	0.97	2.31	0.93	1.04
6	15 µM CLB		0.94	1.52	1.41	1.05	1.71	1.54	1.33	0.75	1.61	0.80	1.33	2.05	1.39	1.56
7	1 µM NU7441		0.86	0.28	1.16	1.40	1.61	1.26	1.62	1.22	1.38	1.09	1.18	0.75	0.84	1.67
8	CLB + NU7441		0.79	0.21	1.13	1.35	1.24	1.22	1.58	1.28	0.85	1.22	0.88	1.87	0.65	1.66

			ATM:Vinc or Actin													
	Patient		1126	257	1410	1089	1262	1441	1387	1191	1173	1093	1413	1406	1172	1309
Well	% Del 11q		0	0	0	0	0	0	0	0	79	85	87	89	98	Del 17
1	DMSO	SFM	1.10	1.06	0.84	1.77	1.21	1.38	2.49	1.50	0.41	1.17	1.42	0.16	1.14	1.17
2	15 µM CLB		0.83	1.36	0.61	1.14	1.19	0.99	0.78	0.96	0.89	1.29	1.41	1.28	1.06	1.07
3	1 µM NU7441		1.11	1.65	1.17	0.96	1.17	1.28	0.91	1.07	1.09	1.32	1.18	1.50	1.22	1.02
4	CLB + NU7441		0.74	0.72	0.72	0.90	1.11	0.79	1.20	0.73	1.24	1.14	1.11	0.48	0.97	0.87
5	DMSO	CD40L/IL4	1.03	0.80	1.67	0.80	0.98	1.22	2.07	1.04	1.56	1.14	1.10	0.92	1.18	0.97
6	15 µM CLB		1.30	0.86	1.20	1.03	1.15	1.26	1.13	0.76	1.25	1.32	1.24	1.20	1.00	1.32
7	1 µM NU7441		0.86	1.13	1.27	1.16	1.40	1.13	1.07	1.11	0.92	1.18	0.76	1.20	0.90	1.08
8	CLB + NU7441		0.85	0.79	0.77	1.02	1.09	1.27	0.90	0.71	0.96	1.08	0.85	0.62	0.58	1.25

			KAP1:Vinc or Actin													
	Patient		1126	257	1410	1089	1262	1441	1387	1191	1173	1093	1413	1406	1172	1309
Well	% Del 11q		0	0	0	0	0	0	0	0	79	85	87	89	98	Del 17
1	DMSO	SFM	0.77	1.13	0.90	1.35	0.98	0.59	1.35	1.38	0.73	0.33	0.93	0.87	1.04	0.43
2	15 µM CLB		0.86	1.40	1.46	1.26	0.97	0.46	1.12	1.18	0.91	0.52	1.29	0.95	1.12	1.01
3	1 µM NU7441		1.18	1.29	1.00	1.25	1.03	0.76	1.24	1.24	0.93	1.03	1.08	0.86	1.30	0.96
4	CLB + NU7441		0.95	0.82	1.06	1.14	0.95	0.73	1.09	1.16	0.98	0.98	1.14	0.87	0.69	1.23
5	DMSO	CD40L/IL4	0.89	2.19	1.22	1.00	0.96	1.06	0.96	0.94	1.13	1.17	1.05	0.94	1.11	1.15
6	15 µM CLB		0.89	0.68	1.15	1.00	1.00	0.92	0.69	0.86	1.11	1.37	1.17	0.84	0.99	1.43
7	1 µM NU7441		1.03	0.74	0.89	1.11	1.14	1.30	0.85	0.84	1.18	1.48	0.90	1.23	0.98	1.75
8	CLB + NU7441		1.04	0.89	0.65	0.90	1.00	1.67	0.81	0.68	1.29	1.40	0.89	1.73	0.85	1.49

Table S4

Lane	Patient	Sex	Age	*IGHV*	FISH (%)	Clinical Treatment (yrs)
P	45	M	59	M	Del 11q (34)	Pre FCR (0.36)
C			68			Post FCR (8.37); On IBR (0.02)
I			69			On IBR (0.23)
P	708	F	68	U	Normal	Pre FCR (0.05)
C			72			Post FCR (3.33); Pre IBR (0.02)
I			72			Post IBR (0.22)
P	929	M	68	M	Del 11q (78), 13q (76)	Pre FCR (0.01)
C			71			Post FCR (2.41); Pre IBR (0.07)
I			71			Post IBR/VEN (0.21)
P	389	M	60	U	Del 11q (85), Tri 12 (94)	Pre FCR (0.13)
C			63			Post FCR (2.33)
P	780	F	67	M	Normal	Pre FCR (0.02)
C			69			Post FCR (1.86)
P	322	M	79	U	Del 13q (80)	Pre CLB/Ob (0.27)
C			81			Post CLB/Ob (1.19); Pre IBR (0.02)
I			81			On IBR (0.22)

P, pre treatment; C, post chemoimmunotherapy; I, on (IBR) ibrutinib; Del, deletion; Tri, trisomy; FCR, fludarabine, cyclophosphamide, rituximab; VEN, venetoclax; CLB, chlorambucil; Ob, obinutuzumab

Table S5

Patient	Sex	Age at sample	*IGHV*	FISH (%)	Time between samples (yrs)	
780	F	64<br>67	M	Normal	2.74	Required Treatment
800	M	62<br>65	M	Tri 12 (73), Del 13q (95)	3.41	Required Treatment
1076	M	52<br>54	U	Del 11q (86), 13q (10)	2.68	Required Treatment
1172	M	61<br>63	U	Del 11q (98), Del 13q (98)	2.12	Required Treatment
795	M	69<br>72	M	Tri 12 (68)	3.39	Required Treatment
896	F	75<br>80	U	Del 13q (10)	4.93	Required Treatment
832	M	67<br>70	M	Del 13q (85)	2.23	Required Treatment
827	M	67<br>71	M	Normal	4.23	Required Treatment
1343	F	68<br>71	N/A	N/A	2.25	Did Not Require Treatment
783	M	64<br>67	M	Tri 12 (46), Del 13q (43)	3.01	Did Not Require Treatment
1050	M	75<br>77	M	Del 11q (38), Del 13q (19)	1.88	Did Not Require Treatment
1106	M	62<br>64	M	Del 13q (91)	2.03	Did Not Require Treatment
760	M	66<br>69	M	Tri 12 (52)	2.97	Did Not Require Treatment
883	M	65<br>70	N/A	N/A	5.21	Did Not Require Treatment
884	F	81<br>85	M	N/A	3.98	Did Not Require Treatment
868	M	64<br>68	M	N/A	3.98	Did Not Require Treatment

U, unmutated; M, mutated; Del, deletion; Tri, trisomy; N/A, not available, yrs, years

Table S6

CLB EC_{50} (µM)

#	Del 11 (%)	SFM DMSO	SFM 1 µM NU7441	CD40L/IL4 DMSO	CD40L/IL4 1 µM NU7441	IgM DMSO	IgM 1 µM NU7441
1*	0	8.6	<2.5	23.3	21.7	8.0	<2.5
2*	0	8.8	<2.5	15.5	7.1		
3*	0	9.3	Note[1]	20.3	3.0	10.1	3.1
4*	0	10.1	Note[1]	31.5	10.0	16.7	3.0
5*	0	10.6	<2.5	15.0	7.9	15.1	<2.5
6*	0	12.0	<2.5	19.6	10.2	14.4	3.5
7*	0	13.9	1.2	15.8	13.0	8.7	1.5
8***	97	14.3	<2.5	26.3	30.8	9.7	<2.5
9*	0	15.3	<2.5	22.0	29.1	11.2	2.8
10**	9.6	16.2	<2.5	22.4	10.5	6.1	<2.5
11*	0	16.3	<2.5	37.4	13.1		
12*	0	16.7	<2.5	8.0	7.1	5.7	<2.5
13*	0	19.5	<2.5	24.4	30.3	6.6	<2.5
14*	0	20.9	<2.5	22.2	13.7	12.0	3.0
15**	18	22.5	7.7	27.0	13.8	15.9	4.5
16**	21	23.2	4.4	>80	>80	22.2	3.5
17*	0	27.3	3.6	40.3	28.6		
18***	85	28.0	7.2	56.4	64.3	15.5	12.7
19*	0	31.2	7.8	58.4	59.5		
20**	27	31.6	2.9	53.2	51.5	23.8	2.6
21*	0	36.6	3.9	29.7	15.3		
22*	0	37.8	5.4	>80	>80		
23***	82	37.8	6.1	27.5	34.0		
24***	66	38.4	5.0	14.7	58.9		
25*	0	39.4	4.5	72.6	67.2	30.6	3.1
26***	87	47.2	25.9	>80	>80	37.7	22.5
27***	79	61.4	30.0	>80	>80	36.0	19.2
28	Del 17p	75.7	28.4	>80	>80	39.3	15.7
Median	Total N=24	21.7	3.3	27.2	28.9	14.8	3.0
	Not Del 11q/17p N=15	15.8	2.5	22.7	13.4	10.7	2.7
	Del 11q N=8	29.8	5.6	40.3	55.2	19.1	4.0
	Del 11q <50% N=3	22.9	3.7	40.1	32.7	19.1	3.1
	Del 11q >50% N=5	38.1	6.7	41.9	61.6	25.8	16.0
Viability of PBMCs N=4		>40	23.5				
% CD19+ cells		25.5	6.8				
Viability of CD19+ cells		>40	28.6				
% CD3+ cells		>40	>40				
Viability of CD3+ cells		>40	>40				

FLU EC_{50} (µM)

#	Del 11 (%)	SFM DMSO	SFM 1 µM NU7441	CD40L/IL4 DMSO	CD40L/IL4 1 µM NU7441	IgM DMSO	IgM 1 µM NU7441
1*	0	1.3	0.9	14.3	>20	1.1	<0.6
2*	0	1.2	0.8	1.8	2.5		
3*	0	1.6	Note[1]	>20	>20	1.0	0.9
4*	0	4.5	Note[1]	9.3	6.1	2.4	1.8
5*	0	0.9	0.8	0.8	<0.6	0.9	0.7
6*	0	1.3	<0.6	1.5	2.9	1.4	0.8
7*	0	<0.6	<0.6	<0.6	1.9	<0.6	<0.6
8***	97	3.8	1.4	9.0	>20	<0.6	<0.6
9*	0	4.6	4.6	16.3	14.3	3.4	3.1
10**	9.6	1.7	1.0	5.8	5.3	0.9	1.0
11*	0	0.8	<0.6	>20	12.6		
12*	0	0.7	<0.6	<0.6	0.9	<0.6	<0.6
13*	0	2.9	0.8	6.0	16.8	0.9	0.6
14*	0	1.7	0.9	0.7	2.4	0.8	0.9
15**	18	>20	12.7	>20	>20	>20	14.8
16**	21	>20	0.8	>20	>20	0.9	0.8
17*	0	2.5	1.1	>20	>20		
18***	85	0.8	<0.6	>20	>20	<0.6	<0.6
19*	0	9.7	3.9	>20	>20		
20**	27	>20	1.6	>20	>20	16.6	1.4
21*	0	10.5	2.5	5.2	2.8		
22*	0	11.6	2.0	>20	>20		
23***	82	6.8	1.5	>20	>20		
24***	66	2.4	<0.6	<0.6	>20		
25*	0	3.5	1.1	>20	>20	3.0	1.0
26***	87	2.8	<0.6	>20	>20	0.7	<0.6
27***	79	8.9	2.2	>20	>20	2.6	0.9
28	Del 17p	>20	>20	>20	>20	>20	>20
Median	Total N=24	2.9	1.0	18.2	20.0	1.0	0.8
	Not Del 11q/17p N=15	1.7	0.8	7.7	9.4	1.0	0.7
	Del 11q N=8	5.3	1.2	20.0	20.0	0.9	0.8
	Del 11q <50% N=3	20.0	1.3	20.0	20.0	8.8	1.2
	Del 11q >50% N=5	3.3	1.0	20.0	20.0	0.7	0.6
Viability of PBMCs N=4		>10	>10				
% CD19+ cells		2.0	2.8				
Viability of CD19+ cells		>10	>10				
% CD3+ cells		>10	>10				
Viability of CD3+ cells		>10	>10				

BEN EC_{50} (µM)

#	Del 11 (%)	SFM DMSO	SFM 1 µM NU7441	CD40L/IL4 DMSO	CD40L/IL4 1 µM NU7441	IgM DMSO	IgM 1 µM NU7441
1*	0	34.1	5.3	96.6	>160	33.2	<5
2*	0	22.2	<5	49.0	19.5		
3*	0	27.4	Note[1]	43.4	17.6	30.5	9.1
4*	0	39.2	Note[1]	57.5	19.0	42.0	6.5
5*	0	15.9	<5	36.6	13.7	28.7	<5
6*	0	Note[2]	Note[2]	25.1	13.8	24.4	<5
7*	0	26.5	<5	44.1	27.6	20.7	<5
8***	97	48.9	9.0	63.7	49.0	22.8	<5
9*	0	13.6	<5	37.7	21.8	13.1	<5
10**	9.6	31.0	6.4	51.3	29.0	22.9	7.2
11*	0	71.0	6.6	145.9	54.5		
12*	0	50.6	6.6	30.1	32.8	37.7	10.0
13*	0	21.1	<5	57.5	14.6	20.3	<5
14*	0	62.7	9.5	77.4	36.9	49.7	13.4
15**	18	51.9	14.9	43.5	23.4	30.2	10.1
16**	21	31.2	6.8	142.5	112.2	35.9	7.5
17*	0	98.6	15.3	>160	104.0		
18***	85	55.2	18.3	100.1	124.2	35.9	32.7
19*	0	77.0	19.6	128.1	74.9		
20**	27	85.1	8.0	100.3	102.1	67.3	8.0
21*	0	118.3	18.4	94.9	40.1		
22*	0	116.9	20.8	>160	>160		
23***	82	78.9	13.7	63.5	57.5		
24***	66	51.6	<5	33.7	43.8		
25*	0	38.0	12.3	104.4	75.0	53.1	7.6
26***	87	90.7	46.6	>160	>160	98.4	47.8
27***	79	73.3	57.1	103.6	118.5	77.3	44.2
28	Del 17p	Note[2]	Note[2]	>160	>160	61.2	28.1
Median	Total N=24	51.1	8.5	70.6	46.4	34.6	7.5
	Not Del 11q/17p N=15	38.0	6.6	57.5	30.2	29.6	5.0
	Del 11q N=8	53.5	11.4	81.9	79.8	35.9	9.1
	Del 11q <50% N=3	41.5	7.4	75.8	65.6	33.0	7.7
	Del 11q >50% N=5	64.2	16.0	81.9	88.0	56.6	38.4
Viability of PBMCs N=4		>80	>80				
% CD19+ cells		43.4	14.5				
Viability of CD19+ cells		58.2	>80				
% CD3+ cells		>80	>80				
Viability of CD3+ cells		>80	>80				

IBR EC_{50} (µM)

#	Del 11 (%)	SFM DMSO	SFM 1 µM NU7441	CD40L/IL4 DMSO	CD40L/IL4 1 µM NU7441	IgM DMSO	IgM 1 µM NU7441
1*	0	3.3	2.9	7.6	8.4	3.2	2.1
2*	0	6.3	8.2	9.6	8.4		
3*	0	<1.3	Note[1]	13.9	11.0	3.7	2.9
4*	0	<1.3	Note[1]	6.3	7.9	3.1	2.4
5*	0	1.7	2.6	7.3	8.9	3.4	2.7
6*	0	3.7	3.2	9.6	9.1	5.7	2.8
7*	0	3.9	3.4	10.0	9.0	3.8	2.9
8***	97	1.5	<1.3	8.8	10.9	9.4	2.6
9*	0	6.2	7.0	10.1	10.4	6.5	5.1
10**	9.6	10.0	9.4	14.8	13.7	15.2	8.6
11*	0	4.8	5.6	11.4	12.2		
12*	0	3.5	4.5	10.6	11.0	7.1	3.8
13*	0	2.1	1.9	10.5	12.0	3.6	3.7
14*	0	4.6	7.2	10.8	11.2	7.2	6.0
15**	18	4.1	5.4	8.9	10.2	6.6	5.8
16**	21	3.1	2.3	10.7	10.3	5.3	2.8
17*	0	3.9	6.5	10.1	9.9		
18***	85	6.6	2.6	18.1	13.3	5.0	4.2
19*	0	6.9	7.7	12.0	13.0		
20**	27	3.4	2.7	6.3	6.2	3.9	2.4
21*	0	7.4	6.4	13.8	13.7		
22*	0	6.9	5.9	16.9	20.0		
23***	82	5.9	4.6	11.3	12.5		
24***	66	5.9	5.2	10.0	10.5		
25*	0	11.2	9.7	14.2	12.3	8.0	8.9
26***	87	11.1	8.5	12.0	10.7	10.8	7.9
27***	79	6.3	6.0	10.1	10.4	7.2	5.0
28	Del 17p	3.5	3.2	9.9	8.4	5.7	3.2
Median	Total N=24	4.4	5.3	10.3	10.6	5.7	3.5
	Not Del 11q/17p N=15	3.9	5.8	10.3	10.7	3.7	2.9
	Del 11q N=8	5.9	4.9	10.4	10.6	6.9	4.6
	Del 11q <50% N=3	3.8	4.0	9.8	10.2	6.0	4.3
	Del 11q >50% N=5	6.1	4.9	10.7	10.8	8.3	4.6
Viability of PBMCs N=4		23.1	22.6				
% CD19+ cells		20.8	26.7				
Viability of CD19+ cells		18.7	17.4				
% CD3+ cells		>40	>40				
Viability of CD3+ cells		23.00	21.7				

IDE EC_{50} (µM)

#	Del 11 (%)	SFM DMSO	SFM 1 µM NU7441	CD40L/IL4 DMSO	CD40L/IL4 1 µM NU7441	IgM DMSO	IgM 1 µM NU7441
1*	0	16.6	31.1	>80	>80	20.5	31.0
2*	0	18.1	64.2	>80	>80		
3*	0	<2.5	Note[1]	>80	>80	9.2	22.8
4*	0	3.6	Note[1]	>80	11.7	>80	10.6
5*	0	6.1	25.1	>80	15.7	>80	45.5
6*	0	17.9	27.6	>80	15.1	>80	15.4
7*	0	24.9	33.0	80.0	20.1	80.0	21.4
8***	97	9.0	16.3	>80	>80	19.8	31.0
9*	0	15.8	41.1	>80	>80	59.0	57.5
10**	9.6	42.6	64.0	>80	>80	>80	>80
11*	0	7.5	15.8	>80	>80		
12*	0	11.1	47.1	>80	42.6	>80	42.4
13*	0	3.9	12.1	>80	>80	15.6	10.5
14*	0	8.9	27.4	>80	27.7	>80	59.2
15**	18	20.1	54.0	>80	>80	42.8	50.2
16**	21	4.7	10.0	>80	>80	6.0	8.0
17*	0	9.2	>80	>80	>80		
18***	85	20.9	11.8	>80	>80	19.1	37.7
19*	0	43.2	78.0	>80	>80		
20**	27	14.7	25.8	>80	29.8	>80	34.6
21*	0	22.0	38.2	>80	>80		
22*	0	54.1	65.5	>80	>80		
23***	82	59.0	64.0	>80	>80		
24***	66	27.0	41.2	>80	>80		
25*	0	30.1	54.2	>80	>80	17.6	19.8
26***	87	>80	>80	>80	>80	>80	>80
27***	79	17.8	43.5	>80	62.1	>80	59.6
28	Del 17p	10.8	36.1	>80	>80	23.2	41.6
Median	Total N=24	17.2	39.6	80.0	80.0	59.0	36.1
	Not Del 11q/17p N=15	13.4	35.6	80.0	80.0	59.0	22.1
	Del 11q N=8	20.5	42.3	80.0	80.0	61.4	43.9
	Del 11q <50% N=3	17.4	39.9	80.0	80.0	61.4	42.4
	Del 11q >50% N=5	23.9	42.3	80.0	80.0	49.9	48.6
Viability of PBMCs N=4							
% CD19+ cells							
Viability of CD19+ cells							
% CD3+ cells							
Viability of CD3+ cells							

*, Not Del 11q or Del 17p; **, <50% Del 11q; ***, >50% Del 11q; Note[1], All cells were killed by 1 µM NU7441 alone; Note[2], the background cell death was too high.

Table S7

Lane	Patient	Sex	Age	*IGHV*	FISH (%)	Clinical Treatment (yrs)
1	171	M	58	U	Del 17p (42), Tri 12 (38)	Post CLB (15.36); Pre IBR (0.02)
2			60			On IBR (1.71)
3	847	M	75	U	Tri 12 (47)	Post FR (2.36), FCR (1.63); Pre IBR (0.02)
4*			78			On IBR (2.55)
5*			79			On IBR (3.66)
6	38	M	70	M	Del 13q (96)	Post FR (2.84); Pre IBR (0.00)
7			71			On IBR (0.86)
8			72			On IBR (2.17)
9	457	F	85	M	Del 17p (89), Tri 12 (84)	Post CLB/Pred (8.62), RCD (8.35), RCD (3.67); Pre IBR (0.02)
10			87			On IBR (2.39)
11	322	M	81	U	Del 13q (80)	Post CLB/Ob (1.19); IBR (0.02)
12			82			On IBR (0.95)
13	309	M	53	U	Normal	Post FCR (5.84); IBR (0.05)
14		M	54			On IBR (0.68)
15		M	56			On IBR (2.29)

*Sample is PBMCs not isolated B cells; U, unmutated; M, mutated; Del, deletion; Tri, trisomy; CLB, chlorambucil; IBR, ibrutinib; FR, fludarabine, rituximab; FCR, FR, cyclophosphamide; Pred, prednisone; RCD, rituximab, cyclophosphamide and dexamethasone

Table S8

Antibody	Species	Dilution	Company	Catalogue #
p-DNA-PK^{S2056}	Rabbit	1:1,000	Abcam	124918
DNA-PK	Mouse	1:1,000	Cell Signaling	12311
p-ATM^{S1981}	Rabbit	1:1,000	Cell Signaling	13050
ATM	Rabbit	1:1,000	Cell Signaling	2873
p-KAP1^{S824}	Rabbit	1:1,000	Cell Signaling	4127
KAP1	Rabbit	1:1,000	Cell Signaling	4124
p-BTK^{Y223}	Rabbit	1:1,000	Cell Signaling	87141
BTK	Rabbit	1:1,000	Cell Signaling	8547
anti-Rabbit	Goat	1:2,000	Bio-Rad	170-6515
anti-Mouse	Goat	1:2,000	Bio-Rad	170-6516
Actin	Rabbit	1:2,000	Sigma	A2066
Vinculin	Mouse	1:10,000	Abcam	ab18058

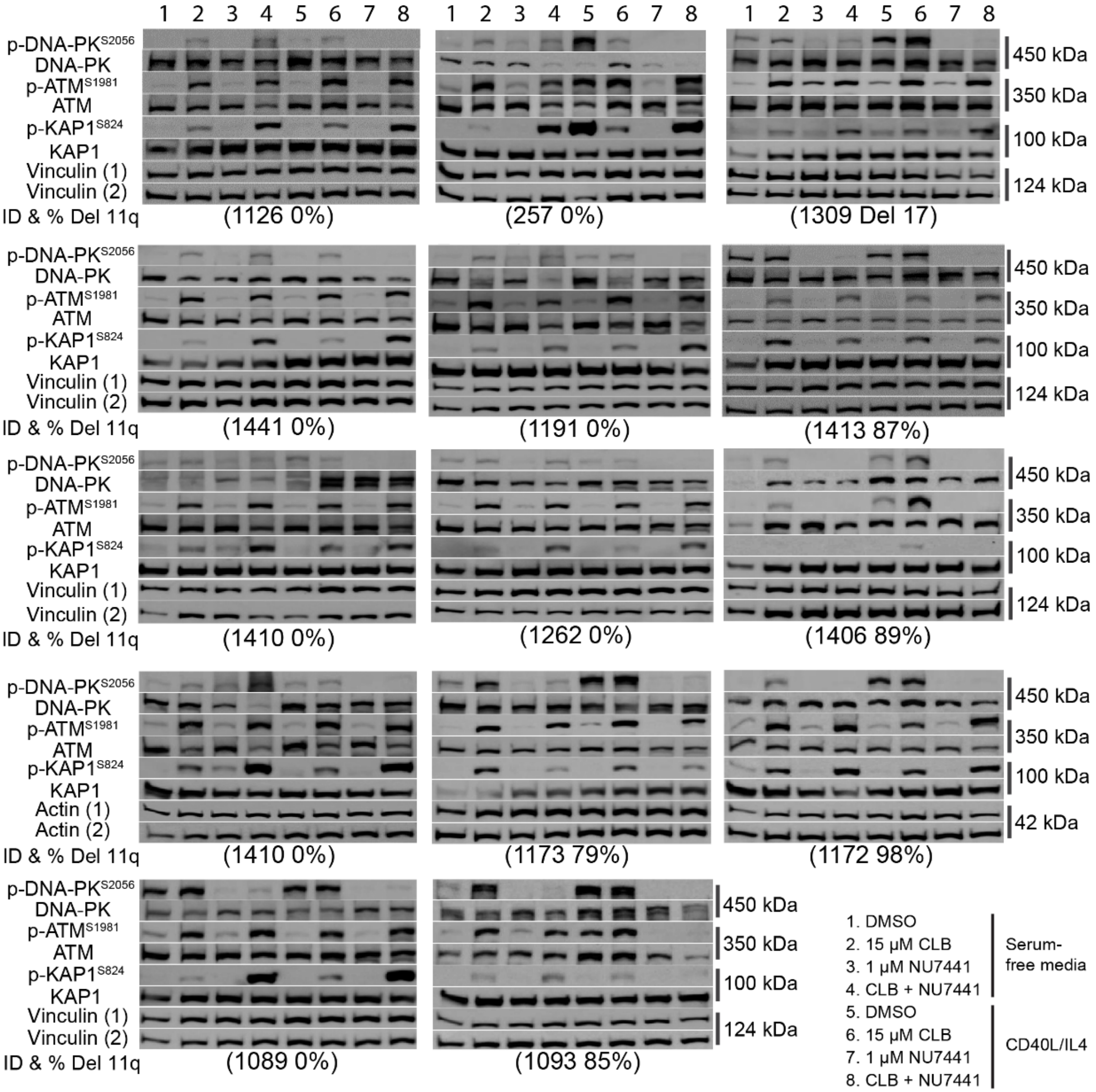

1 2 3 4 5 6 7 8
p-DNA-PK^{S2056}
DNA-PK
p-ATM^{S1981}
ATM
p-KAP1^{S824}
KAP1
Vinculin (1)
Vinculin (2)
Actin (1)
Actin (2)
ID & % Del 11q
(1126 0%)
(257 0%)
(1309 Del 17)
(1441 0%)
(1191 0%)
(1413 87%)
(1410 0%)
(1262 0%)
(1406 89%)
(1410 0%)
(1173 79%)
(1172 98%)
(1089 0%)
(1093 85%)
450 kDa
350 kDa
100 kDa
124 kDa
42 kDa
1. DMSO
2. 15 μM CLB
3. 1 μM NU7441
4. CLB + NU7441
Serum-free media
5. DMSO
6. 15 μM CLB
7. 1 μM NU7441
8. CLB + NU7441
CD40L/IL4

Figure S1

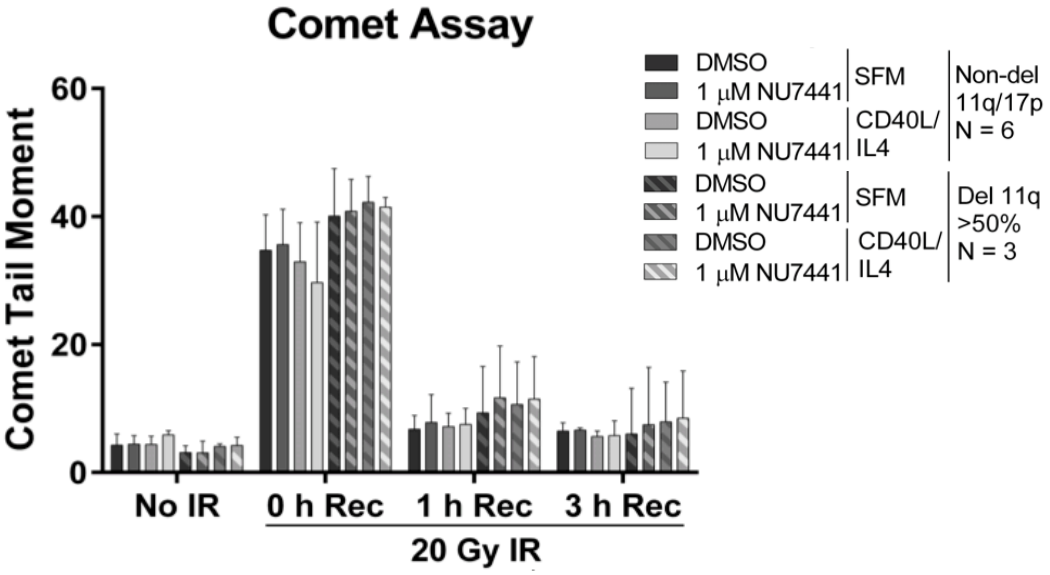
Comet Assay
Comet Tail Moment
60
40
20
0
No IR
0 h Rec
1 h Rec
3 h Rec
20 Gy IR
DMSO
1 μM NU7441
SFM
DMSO
1 μM NU7441
CD40L/ IL4
Non-del 11q/17p N = 6
DMSO
1 μM NU7441
SFM
DMSO
1 μM NU7441
CD40L/ IL4
Del 11q >50% N = 3

Figure S2

A

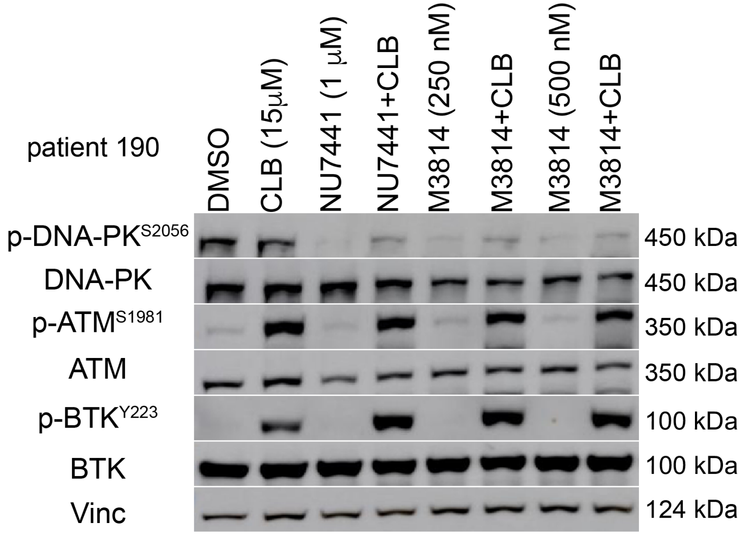

B

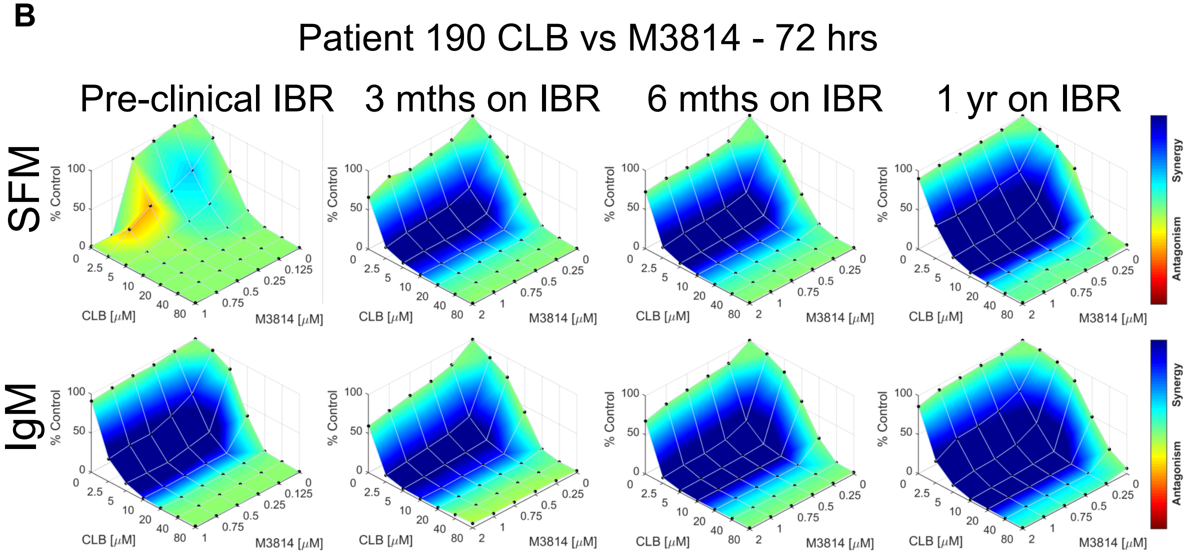

C

Sample	Clinical Information					EC50 CLB (μM)									
						SFM					IgM				
						NU7441 (μM)		M3814 (μM)			NU7441 (μM)		M3814 (μM)		
	Sex	Age	*IGHV*	FISH (%)	Treatment (yrs)	0	1	0	0.25	0.5	0	1	0	0.25	0.5
190	F	81	M	Del 13q (78)	Post CLB (17.73); Pre IBR (0.24)	6.04	<2.5	4.99	<2.5	<2.5	10.68	2.53	8.45	<2.5	<2.5
					On IBR (0.24)	4.86	<2.5	7.25	<2.5	<2.5	8.22	<2.5	8.16	<2.5	<2.5
		82			On IBR (0.47)	6.56	<2.5	6.78	<2.5	<2.5	11.25	<2.5	11.17	<2.5	<2.5
					On IBR (0.96)	10.83	<2.5	12.22	2.95	2.64	20.45	<2.5	20.03	3.37	2.58

Figure S3

A

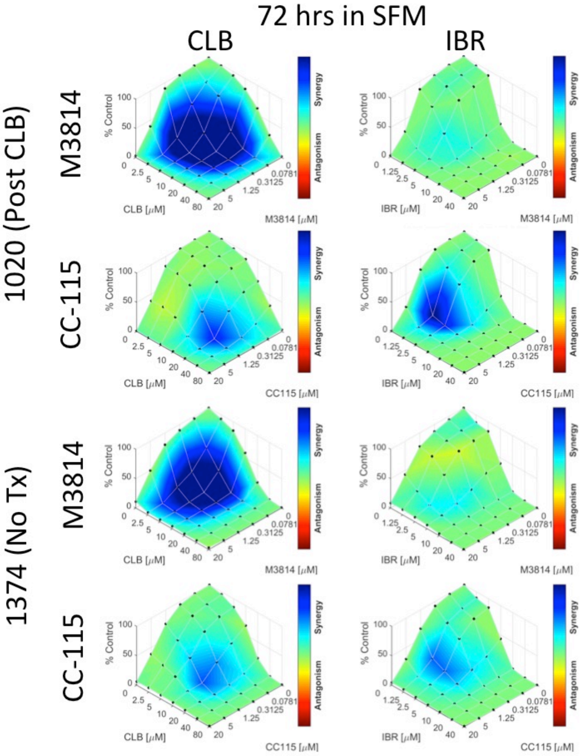

B

Sample	Clinical Information					SFM											
						CLB EC_{50} (μM)						IBR EC_{50} (μM)					
						NU7441		M3814		CC115		NU7441		M3814		CC115	
	Sex	Age	*IGHV*	FISH (%)	Treatment (yrs)	DMSO	1 μM	DMSO	0.3 μM	DMSO	0.3 μM	DMSO	1 μM	DMSO	0.3 μM	DMSO	0.3 μM
1020	M	87	U	Del 11q (82), 13q (81)	Post CLB (2.12), Ob (1.64)	22.28	1.88	23.60	3.10	26.24	16.87	3.87	2.36	3.87	3.24	4.03	2.74
1374	M	84	U	N/A	None	12.19	0.57	11.25	1.97	12.45	7.65	4.22	3.19	4.30	4.01	4.48	2.98

Figure S4

A

Sample	Clinical Information					EC50 CLB (µM)										EC50 IBR (µM)									
						SFM					IgM					SFM					IgM				
						NU7441 (µM)		M3814 (µM)			NU7441 (µM)		M3814 (µM)			NU7441 (µM)		M3814 (µM)			NU7441 (µM)		M3814 (µM)		
	Sex	Age	*IGHV*	FISH (%)	Treatment (yrs)	0	1	0	0.25	0.5	0	1	0	0.25	0.5	0	1	0	0.25	0.5	0	1	0	0.25	0.5
1301	F	54	M	Tri 12 (3)	None	3.21	<2.5	4.13	<2.5	<2.5	2.87	<2.5	3.24	<2.5	<2.5	4.92	10.24	4.78	8.64	9.73	10.22	9.92	9.48	9.94	9.96
664	F	85	M	Del 13q (8)	None			6.25	<2.5	<2.5			5.87	<2.5	<2.5			9.57	10.00	9.88			9.65	9.71	9.78
368	M	65	M	Del 13q (93)	None	4.64	<2.5	6.46	<2.5	<2.5	4.37	<2.5	6.18	<2.5	<2.5	5.50	6.35	4.66	5.27	5.16	4.49	4.80	4.34	5.00	4.99
651	F	92	M	Del 13q (91)	None			6.88	3.28	2.65			10.36	2.79	<2.5			4.84	6.00	5.59			5.40	5.51	5.94
897	M	69	U	N/A	None			8.97	3.00	3.07			10.48	2.59	<2.5			2.27	5.12	4.51			5.87	5.88	5.81
1340	M	56	M	Del 13q (94)	None	9.25	<2.5	10.02	2.52	<2.5	7.72	<2.5	6.61	<2.5	<2.5	6.48	7.22	6.28	6.35	6.00	6.91	6.66	7.01	6.92	6.04
1230	F	71	M	Normal	None			10.45	<2.5	<2.5			9.32	<2.5	<2.5			5.73	5.51	5.34			5.92	5.81	5.02
1244	M	70	U	Tri 12 (55)	None	12.13	<2.5	13.00	<2.5	<2.5	7.72	<2.5	7.87	<2.5	<2.5	<1.25	3.22	<1.25	<1.25	3.10	5.10	2.16	5.59	4.82	3.73
1041	M	82	U	Del 13q (74)	None	28.77	Dead	25.29	5.75	3.45	15.74	3.20	13.87	5.39	3.25	5.94	5.77	5.83	5.87	5.19	6.44	7.15	6.02	7.04	5.85
1079	F	74	U	Del 11q (50), 13q (19)	None			30.13	3.09	<2.5			10.81	<2.5	<2.5			2.86	2.26	1.90			3.38	2.71	2.79
568	F	73	M	Normal	None	31.33	<2.5	31.70	2.82	<2.5	10.60	<2.5	10.62	<2.5	<2.5	5.61	6.04	5.54	5.47	5.28	5.42	5.81	5.52	5.55	5.40
1356	M	66	M	Del 11q (74)	None			31.92	3.35	<2.5			20.23	3.20	<2.5			5.94	5.90	5.25			7.25	6.56	6.31
1413	F	74	U	Del 11q (87), 13q (83)	None	47.17	25.92	36.46	20.35	18.04	37.70	22.55	26.42	16.21	15.13	11.09	8.50	9.74	10.79	10.96	10.79	7.94	10.14	10.61	10.62
1173	F	73	U	Del 11q (79)	None			36.90	20.33	18.78			19.25	14.20	13.55			3.58	4.05	3.16			4.27	4.99	3.76
1362	F	72	U	Del 17p (79), 13q (80)	None			>80	>80	>80			>80	>80	>80			11.68	11.73	11.57			12.14	12.36	12.12
1020	M	84	U	Del 11q (82), 13q (81)	Pre CLB (0.01)	37.76	6.13									5.87	4.62								
					On CLB (0.22)	>80	4.23									4.39	2.97								
733	F	89	M	Del 13q (82)	Pre IBR (0.00)	15.28	<2.5				11.22	2.85				6.24	7.03				6.53	5.06			
					On IBR (0.70)	7.50	<2.5				8.92	<2.5				6.14	6.22				6.51	5.23			
1440	M	68	U	Del 11q (27)	Pre IBR (0.09)	31.55	2.92				23.77	2.62				3.40	2.66				14.69	25.82			
		69			On IBR (0.55)	65.92	4.72									8.6	5.7								
					On IBR (1.05)	39.54	<2.5				17.62	<2.5				10.72	9.21				>40	>40			
190	F	81	M	Del 13q (78)	Post CLB (17.73); Pre IBR (0.24)	6.04	<2.5	4.99	<2.5	<2.5	10.68	2.53	8.45	<2.5	<2.5										
					On IBR (0.24)	4.86	<2.5	7.25	<2.5	<2.5	8.22	<2.5	8.16	<2.5	<2.5										
		82			On IBR (0.47)	6.56	<2.5	6.78	<2.5	<2.5	11.25	<2.5	11.17	<2.5	<2.5										
					On IBR (0.96)	10.83	<2.5	12.22	2.95	2.64	20.45	<2.5	20.03	3.37	2.58										
171	M	60	U	Del 17p (42), Tri 12 (38)	Post CLB (17.52); On IBR (2.15)	>80	49.17	>80	55.45	50.23	>80	66.83	>80	77.09	48.75	6.81	4.74	6.89	6.80	6.75	7.61	5.72	8.16	8.49	7.99
847	M	80	U	Tri 12 (47)	Post FR (6.77), FCR (6.04); On IBR (4.39)	17.70	<2.5	18.88	4.24	2.75	>80	**7.06**	30.76	7.08	4.10	3.85	4.56	3.45	3.33	2.98	6.16	4.85	6.40	5.77	5.64
505	M	65	U	Normal	Post CLB (14.70), FLU (13.81, 11.98), FCR (10.67,6.18); On IBR (6.08)	55.72	3.46	65.16	5.41	4.09	25.12	3.30	28.64	3.39	<2.5	5.37	4.93	5.44	4.78	4.20	5.98	4.60	6.15	5.75	5.50
830	M	72	M	Del 13q (38)	Post FLU (9.96, 7.91), FCR (7.27); On IBR/Acala (4.42)	>80	69.82	>80	>80	>80	>80	>80	>80	>80	>80	3.87	2.81	3.66	3.89	3.89	6.08	3.94	6.19	5.38	5.29

B

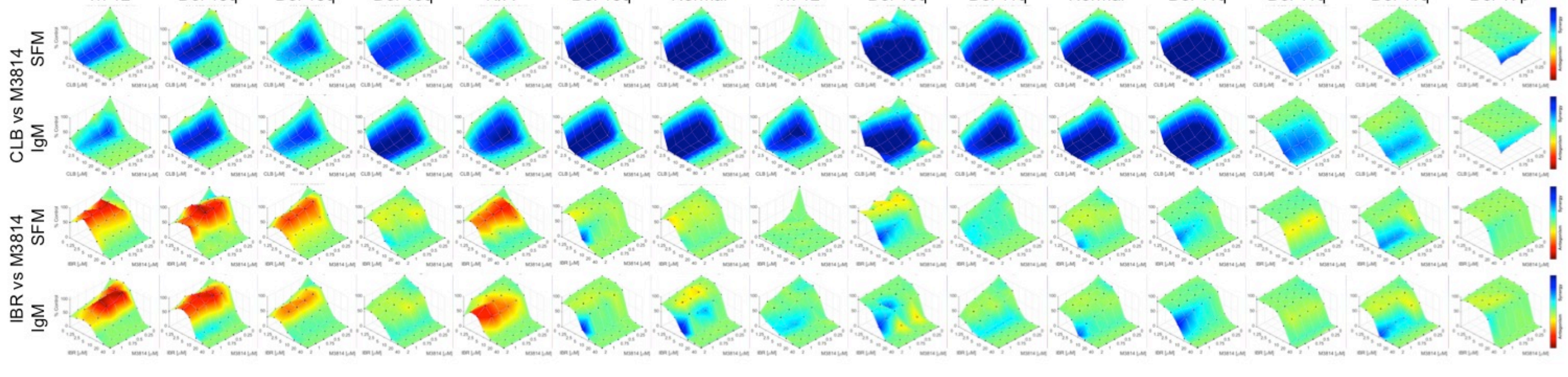

Figure S5
